# Studying the Influence of Apple Peel Polyphenol Extract Fortification on the Characteristics of Probiotic Yoghurt

**DOI:** 10.3390/plants9010077

**Published:** 2020-01-07

**Authors:** Ishtiaque Ahmad, Anjum Khalique, Muhammad Qamar Shahid, Abdul Ahid Rashid, Furukh Faiz, Muhammad Asim Ikram, Sheraz Ahmed, Muhammad Imran, Muhammad Asif Khan, Muhammad Nadeem, Muhammad Inam Afzal, Muhammad Umer, Imdad Kaleem, Muhammad Shahbaz, Bilal Rasool

**Affiliations:** 1 Department of Dairy Technology, University of Veterinary and Animal Sciences, Lahore 54000, Pakistan; ishtiaque@uvas.edu.pk (I.A.); asim.ikram@uvas.edu.pk (M.A.I.); 2 Department of Animal Nutrition, University of Veterinary and Animal Sciences, Lahore 54000, Pakistan; akhalique@uvas.edu.pk; 3 Department of Livestock Production, University of Veterinary and Animal Sciences, Lahore 54000, Pakistan; qamar.shahid@uvas.edu.pk; 4 Food and Biotechnology Research Centre, Pakistan Council of Scientific and Industrial Research, Lahore 54000, Pakistan; ch.abdulahid@gmail.com; 5 Department of Agriculture and Food Technology, Karakoram International University, Gilgit 15100, Pakistan; furrukhfaiz@kiu.edu.pk; 6 Department of Food Sciences, Faculty of Biosciences, Cholistan University of Veterinary & Animal Sciences, Bahawalpur 63100, Pakistan; drsherazahmedft@gmail.com; 7 University Institute of Diet and Nutritional Sciences, Faculty of Allied Health Sciences, The University of Lahore, Lahore 54000, Pakistan; mic_1661@yahoo.com; 8 University of Agriculture, Faisalabad, Sub-Campus Burewala, Vehari 61100, Pakistan; asifkhan.muhammad@gmail.com; 9 Department of Environmental Sciences, COMSATS University Islamabad, Vehari Campus 61100, Pakistan; nadeem@cuivehari.edu.pk; 10 Department of Biosciences, COMSATS University Islamabad, Park Road, Tarlai Kalan, Islamabad 45550, Pakistan; dr-miafzal@hotmail.com (M.I.A.); umer.imperial@gmail.com (M.U.); kaleemgcs@gmail.com (K.I.); 11 Department of Food Science and Technology, MNS-University of Agriculture, Multan 66000, Pakistan; 12 Department of Zoology, Faculty of Life Sciences, Government College University Faisalabad, Punjab 38000, Pakistan

**Keywords:** by-product, yoghurt, viability, bioactive, viscosity, pulp

## Abstract

The aim of the current study was to evaluate the effect of apple peel polyphenol extract (APPE) on the physicochemical and microbiological properties of probiotic yoghurt. Five concentrations of APPE were added in probiotic yoghurt as: (1) CTL, control without APPE; (2) AE1, addition of 1% APPE; (3) AE2, addition of 2% APPE; (4) AE3, addition of 3% APPE; (5) AE4, addition of 4% APPE; and (6) AE5, addition of 5% APPE. The prepared probiotic yoghurt was stored at 4 °C for 21 days and analyzed for physicochemical and microbiological properties. The initial viable count of *L. bulgaricus*, *S. thermophilus*, *B. lactis* and *L. acidophilus* were similar in all yoghurt samples at day 1. The maximum viability loss of probiotics was observed in CTL (*p* < 0.05). The lowest viability loss of probiotics was observed in AE5 samples (*p* < 0.05). The acidity, water holding capacity and viscosity were increased with the addition of APPE. No significant effects were observed on milk fat and total solid contents of probiotic yoghurt with the addition of APPE. The total phenolic contents of probiotic yoghurt increased significantly as 0.59, 0.71, 0.97, 1.18, 1.35 in AE1, AE2, AE3, AE4 and AE5, samples respectively. It was observed that AE3 and AE4 samples had better taste, flavour and colour with good texture. The survival of probiotics and antioxidant activity of the yoghurts were enhanced with the addition of APPE. In conclusion, apple peels could be successfully used as prebiotic in yoghurt with increased viable counts of probiotics.

## 1. Introduction

Functional yoghurt products involve the inclusion of probiotics, prebiotics or a mixture of both [1]. Probiotics are live microbial foods having therapeutic impacts on the gastrointestinal tract including prevention of infections, improvement of lactose intolerance, decrease in cholesterol, and modulation of the immunity [2]. Several species of *Lactobacilli* and *Bifidobacteria* are expressed in over ninety percent of probiotic items and popular among health-conscious societies [3]. 

The several bioactive elements from natural sources have nutritional benefits, sensual attributes, physiochemical properties and therapeutic values [4]. Polyphenols (PPs) are well known bioactive antioxidants that could have a positive effect on preventing oxidation [5]. PPs are not only useful for milk products during their shelf life but also beneficial as antioxidant for the human body [6]. Apple ranks 2nd in overall Polyphenol (PP) percentage and has the maximum shear of free PPs compared to other fruits [7]. The total antioxidant capacity of apple peels was about 2.5 times higher than that of apple pulp. About 46% of the polyphenolics are from the peel of the apple as reported by [8]. Utilization of PPs from apple peels offer a substitute for its consumption as an effective prebiotic component. Naturally apple peels contain all the flavonols (quercetin derivatives) which have potential antioxidant activity [8].

In Pakistan, the total production of apple during 2018–2019 was about 565,000 tones [9]. Apples are the 4th most widely consumed fruit in Pakistan [10]. Apple pomace contains peel, flesh, stem, core, seeds and juice residues. A sample of apple pomace was reported to contain 54% pulp, 34% peel, 7% seeds, 4% seed core and 2% stem [11]. The apple peels, although nutritionally important, are often discarded during the processing of apple jam, dried apples, canned apples, and apple pulps in Pakistan. This might be due to the lack of consumer’s awareness and commercial applications of the benefits of apple peels. The viable count of probiotic microbes decreases in acidic milk products during processing, handling and storage, owing to the acidic pH and oxidative stress. The low viability of probiotic bacteria subsequently affects its ability to impart their desired health benefits [12]. Probiotic microbes coated with PPs could be equipped with oxidant-scavenging capacity [13]. Probiotic milk products supplemented with apple PPs may offer a chance to deliver functional assistances to enhancing the viability of probiotics [14]. The objective of this study was to evaluate the effect of apple peel PP on viability of probiotics in yoghurt. PPs may be helping to maintain the functionality and viability of probiotics throughout the shelf life of yoghurt. Besides, the use of the apple peel helps to decrease the total volume of waste created by the apple processing. This aligns with the industrial waste reuse strategy, which suggests the important practical influence of this current study.

## 2. Materials and Methods

This experiment was conducted in the microbiological and physicochemical laboratory, Department of Dairy Technology, University of Veterinary and Animal Sciences (UVAS, Lahore, Pakistan). Buffalo milk was obtained from the UVAS Dairy Farm. The yoghurt starter culture (*Lactobacillus delbrueckii* subsp. *bulgaricus* and *Streptococcus salivarius* subsp. *thermophilus*) and probiotics strains (*Bifidobacterium lactis* BB-12 and *Lactobacillus acidophilus* La-5) was obtained from Sacco (Sacco, Italy). All these cultures were kept in a freezer at −20 °C.

### 2.1. Apple Peel Polyphenol Extract

The extraction of PPs was done as per the method described by Sun-Waterhouse et al. [15]. The commercially available apple varieties Red Delicious (Tur-kulu), Golden Delicious (Shin-kulu), Royal Gala, Amri, and Gacha were obtained from a local market. The extraction of PPs was conducted in the PCSIR laboratories (Lahore, Pakistan). The apples were peeled and the peels were frozen at −80 °C in an ultra-freezer for 2 h. The frozen peel was freeze dried by a FD-550 freeze dryer (EYELA, Tokyo, Japan) at −30 °C with a vacuum of 6.67 Pa. The dried peels were ground into a fine powder by a ring grinder (Rocklabs Bench Top Ring Mill, Christchurch, New Zealand) and stored at −80 °C in an ultra-freezer. The extraction medium was prepared by using acidified water. Acidic solution was arranged by mixing a citric acid in deionized distilled water until the solution reached a pH of 3. Extractions were performed by mixing 1 g thawed freeze-dried apple peel powder with 10 mL of acidified solution before vortexing (2 min). The mixture was centrifuged on a benchtop Universal 32R centrifuge (Hettich Zentrifugen, Tuttlingen, Germany) for 180 s twice at 20 °C. The supernatant was collected, dried by the EYELA FD-550 freeze dryer at −30 °C and stored at −80 °C in ultra-freezer until required.

### 2.2. Quality Characteristics of APPE

Quality characteristics of APPE of the five studied local cultivars (Red Delicious, Golden Delicious, Royal Gala, Amri, and Gacha) were evaluated on the basis of total flavonoid content (TFC), total phenolic contents (TPC) and antioxidant activity.

### 2.3. TPC and TFC of APPE

A solid phase extraction (SPE) column was used to pre-treat prior to the total extractable polyphenolic contents analysis to remove the possible interference of sucrose and vitamin C with the Folin-Ciocalteu reagent. The total phenolic content (0.05 mL) of apple peels was measured by the colorimetric method of Folin-Ciocalteu [16]. Absorbance of UV-Vis spectrophotometer was noted at 765 nm. The quantity of total water-soluble phenolic compounds was determined by a standard curve of water solutions of gallic acid (1 to 10 ppm) and represented as gallic acid equivalents (GAE) g/100 g of extract on dry weight bases. Total flavonoid content (1 mL) of apple peels was determined by the method of colorimetric with aluminum chloride [16]. TFC was measured from a quercetin (1 to 60 ppm) standardization curve, and represented as quercetin equivalents (QE) g/100 g DW of extract.

### 2.4. Preparation of Probiotic Yoghurt

Probiotic yoghurt was prepared with different treatments of apple peel polyphenol extract (APPE) along with a control. The buffalo milk was standardized at 11% solids non-fat and 4% fat content. Milk was homogenized and pasteurized at 85 °C for half hour [17]. The milk was cooled at 42 °C and divided into six parts. One was a control (CTL) and the other five parts were mixed with different concentrations of APPE 1%, 2%, 3%, 4% and 5% (AE1, AE2, AE3, AE4, and AE5) to make probiotic yoghurt. Samples were incubated with yoghurt starter culture (*L. bulgaricus* and *S. thermophilus*) and probiotic strains (*L. acidophilus* and *Bifidobacterium lactis)* according to company (Sacco) description, each in cleaned sterilized plastic cups at 42 °C in incubator until the required acidity (pH 4.6). All yoghurt samples were stored at 4 °C and evaluated after 1, 7, 14, and 21 days. Every treatment was performed in triplicate.

### 2.5. Microbiological Analysis

The viable count of yoghurt starter culture and probiotic microbes was performed after 1, 7, 14 and 21 days. The sample and dilution preparation for viable count and microbiological inspection was performed according to IDF standard [18]. The count of live bacteria in yoghurt samples was measured by tenfold serial dilution and pour plate technique. The number of *L. acidophilus* and *B. lactis* strains were incubated on selective de man rogosa and sharpe (MRS) agar with sorbitol and MRS-NNLP (nalidixic acid, neomycin sulphate, lithium chloride, and paramomycin sulphate) agar, respectively. The viability of *S. thermophilus* was determined by a selective media of M17 agar. The media plates kept in aerobic conditions at 37 °C for two days. The selective media reinforced clostridia agar (RCA) used for *L. bulgaricus* under anaerobic conditions for two days at 42 °C [19].

### 2.6. Physicochemical Analysis

Total solids (TS), milk fat, acidity and pH were measured according to the methods proposed by Association of Official Analytical Chemists [20]. TS contents were analysed by dry oven method. Fat contents were measured by Gerber method. The pH values of yoghurt were determined by a pH meter (Hanna Instruments; model pH 211). Acidity was determined by acid base titration. A 10 g sample mixed with 10 mL of hot distilled water and titrated against 0.1N sodium hydroxide with 0.5% phenolphthalein as an indicator. Analysis were performed in triplicate after 1, 7, 14, and 21 days at 4 °C.

The viscosity of the yoghurt was calculated, after mixing the sample for 60 s, with the help of a model DV2T viscometer (Brookfield, WI, USA) at 4 °C. Samples were analysed using spindle number 4 at 12 rpm and data were recorded in centipoise (Cp) as duplicate [21]. The water holding capacity (WHC) was measured through an adapted technique by Remeuf et al. [22]. A 20 g yoghurt sample was centrifuged at 5000 *g* for 10 min at 20 °C. The expelled whey (EW) drained and sample weighed as grams. The WHC was calculated as:

$$\mathrm{WHC}\;\left(\%\right)=\frac{\left(\mathrm{Sample}-\mathrm{EW}\right)}{\left(\mathrm{Sample}\right)}\times 100$$

Total phenolic contents (TPC) (0.05 mL) was measured according to the Folin Ciocalteau colorimetric procedure as adopted by Moo-Huchin et al. [16]. Absorbance read at 765 nm with a spectrophotometer (Lambda 11, Perkin Elmer, Waltham, Massachusetts, United States). A standard curve of aqueous solutions of gallic acid (1–10 ppm) utilized to calculate the quantity of total polyphenolic contents and its unit is mg gallic acid equivalents (GAE)/100 g dry weight of sample. The total flavonoid content (TFC) (1 mL) was measured by the method of aluminium chloride colorimetric adopted by Moo-Huchin et al. [16]. A quercetin (1–60 ppm) calibration curve used to calculate TFC, and its unit was g of quercetin equivalents/100 g (g QE/100 g DW). DPPH free radical scavenging activity was measured by the procedure as adopted by Khanahmadi et al. [23]. DPPH (0.1 mM) solution was prepared in methanol. A 0.1 mL sample mixed with 2.9 mL DPPH in screw capped test tube and vortexed at 500 × *g* for 120 s, followed by incubation for 30 min. Absorbance recorded at 517 nm in visible section of spectrum. The antioxidant activity was measured by calculating the free radical scavenging ability of probiotic yoghurt with DPPH inhibition.

### 2.7. Sensory Evaluation

Sensory quality evaluation for the probiotic yoghurt was done using a descriptive test. Ten trained panellists from the Central Lab Complex, Department of Dairy Technology, (UVAS, Lahore, Pakistan) participated in the evaluation; all had experience of standardized tests for olfactory and taste sensitivities as well as verbal abilities and creativity. They evaluated 30 g portions of each probiotic yoghurt sample and used a quality rating score card for evaluation of colour (nine points), flavour (nine points), taste (nine points) and texture (nine points) as described by Rogers [24]; The highest number indicating extreme liking and the lowest extreme dislike. Each sample was recorded separately, and the samples delivered to the panellists in the isolated containers. Samples of control and yoghurt prepared with 1%, 2%, 3%, 4% and 5% APPE were coded with three numbers, and delivered to the panellists randomly at every session. Sensory characteristics of the probiotic yoghurt were valued by panellists after 1, 7, 14 and 21 days.

### 2.8. Data Analysis

Data analysis were performed by two-way analysis of variance technique using the SAS^®^ 9.1 software [25]. The difference in mean values of two factors were analyzed at significance level of 0.05 using least significant difference test.

## 3. Results and Discussion

### 3.1. Quality Characteristics of APPE

**Figure 1:**
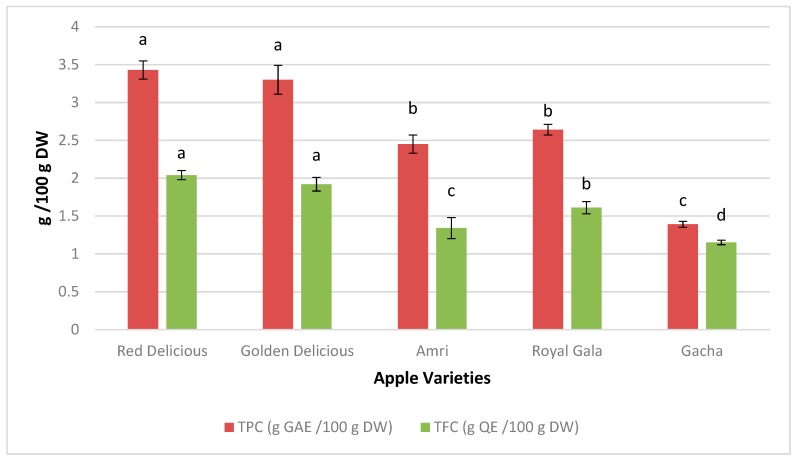
Total phenolic contents and total flavonoid contents of APPE of different apple varieties. Results presented by the average of the triplicates followed ± standard deviation. Different letters in a column of same color denote significant differences (*p* ≤ 0.05). APPE: Apple peel polyphenol extract; GAE: gallic acid equivalents; QE: quercetin equivalents.

The most common naturally available phenolics are flavonoids due to their wide range of biological and biochemical activities. The real antioxidant potential is often more precisely exposed by presenting the activity of antioxidant in forms of total flavonoids and phenolics. TPC and TFC in APPE of different apple cultivars are shown in Figure 1. The total phenolic content has a significant variation between the APPE of local apple varieties. The maximum total phenolic content (3.43 g GAE/100 g dry weight) were found in APPE of ‘red delicious’, followed by ‘Golden Delicious’, ‘Royal Gala’, ‘amri’, and ‘gacha’, which had (3.30, 2.64, 2.45, 1.39 g GAE/100 g dry weight). The total soluble flavonoid contents, between the APPE of five apple cultivars ranged from 2.04 to 1.15 g QE/100 g of apple peel extract (Figure 1). The APPE of red delicious revealed maximum (2.04 g QE/100 g dry weight) flavonoid contents significantly followed by Golden Delicious, Royal Gala, amri, and gacha, (1.94, 1.61, 1.34, 1.15 g QE/100 g dry weight). Can-Cauich et al. [26] examined that peel extracts of 11 tropical fruits were found to have high antioxidant activity and high bioactive compound contents. The total flavonoids, total phenolics and antioxidant activity values were found significantly high in purple sugar apple and green sugar apple. Generally, it was observed that apple peel possesses high phenolics and flavonoids contents as compared to the fruit pulp, it is due to the nature and scattering of flavonoids and phenolics varies inside the different portions of a same fruit.

### 3.2. Microbiological Characteristics of Probiotic Yoghurt

Outcomes of all microbiological characteristics in probiotic yoghurt throughout 21 days at 4 °C presented significant effects (*p* < 0.05). The initial viable counts of *L. bulgaricus*, *S. thermophilus*, *B. lactis* and *L. acidophilus* were similar in all yoghurt samples, which revealed non-significant effects (*p* > 0.05) at day 1 (Table 1). It was observed that the viability of all four bacteria decreased significantly in control yoghurt samples during storage (*p* < 0.05). The results revealed that at day 1, CTL had the viable count of *S. thermophilus* 10.63 log cycle, after 21 days the loss in viability was 2.85 log cycle, whereas with APPE 1%, 2%, 3%, 4%, and 5% fortification these losses were 1.67, 1.48, 1,24, 0.79 and 0.47 log cycles, respectively after 21 days. The results revealed that AE1 had less loss of *S. thermophilus* 1.67, followed by AE2 0.47 log cycle, respectively after 21 days. The initial count of *L. bulgaricus* was 10.24 log cycle and after 21 days the loss in viability was 2.64 log cycle in control sample whereas with APPE 1%, 2%, 3%, 4%, and 5% fortification these losses were 1.90, 1.25, 1.05, 0.74 and 0.36 log cycles, respectively. In the former study [27], the APPE enhanced the viability of *Streptococcus* and *Lactobacillus* in yoghurt. Some unfavorable impacts of phlorizin and chlorogenic acid on *Lactobacillus* were observed. Addition of APPE before incubation found in a more uniform distribution of *Lactobacillus* and *Streptococcus* as well as uneven distribution was reported in the control. 

The initial count of *L. acidophilus* in control yoghurt samples was 11.14 log cycle and after 21 days the loss in viability was 3.07 log cycle whereas with APPE 1%, 2%, 3%, 4%, and 5% fortification these were 2.24, 2.07, 1.60, 1.08, and 0.75 log cycle, respectively. The initial count of *B. lactis* was 10.71 log cycle and after 21 days the viability loss was 2.90 log cycle in control sample whereas with APPE 1%, 2%, 3%, 4%, and 5% fortification, these were 1.49, 1.15, 0.81, 0.71, 0.57 log cycle, respectively. The lowest viability loss of probiotic bacteria was detected in yoghurt samples with fortification of APPE at 5% significantly (*p* < 0.05). The maximum viability loss of the four microbes were observed in control yoghurt sample for 21 days significantly (*p* < 0.05). The slight decrease of viable count was perceived in all yoghurt samples with fortification of APE during refrigerated conditions. It was previously studied that, the addition of APPE had a significantly positive impact on the viability of *B. lactis* and *L acidophilus* strains [15]. APPE stimulated the survival of *L. acidophilus* more than either of the tested polyphenols, except for rutin. 

These outcomes confirm the potential use of APPE as a source of polyphenols to boost the functionality of probiotics in dairy products furthermore to add value to this waste stream agro-industrial material [28]. Nutritional components of fruits, such as phenolic contents and organic acids, used as a natural energy source for probiotics; the water-soluble food dietary fibers found in plant tissues had a prebiotic impact [29,30]. The results revealed that the utilization of APPE as a prebiotic in yoghurt enhanced the viability of probiotics as compared to control (without APPE) yoghurt. It is recommended that the minimum number of probiotics should be 8 log CFU (colony forming unit)/mL of product per day, otherwise at the time consumption probiotic foods must hold a sufficient quantity of live cells at least 6 log CFU/mL to guarantee their useful impacts [31].

### 3.3. Chemical Characteristics of Probiotic Yoghurt

Milk fat and total solid contents of fresh buffalo’s milk used in probiotic yoghurt manufacturing were; 4% and 15%, respectively after standardization. Results revealed that milk fat of all yoghurt samples were found to be the same levels and non-significant differences observed for 21 days within treatments (*p* > 0.05). Yoghurt samples with APPE had slightly high levels of total solid contents than the control. The results revealed that CTL had the low TS contents 14.44%, followed by AE1 14.56% while, AE5 had the high TS contents 14.96%, then non-significant difference observed during storage (Table 2). 

It was observed that addition of APPE in yoghurt reduced the syneresis while increased firmness and viscosity. The average composition of probiotic yoghurts revealed that the fortification with APPE had non-significant difference (*p* > 0.05) in TS and milk fat contents among yoghurt samples. It was observed that at day 1, CTL had the high pH values 4.56, followed by AE1 4.51 while, AE5 had the low pH 4.36, then significantly (*p* < 0.05) decreased to 4.19, 4.13 and 4.02, respectively, for 21 days. The results revealed that the APPE slightly decreased the pH values of yoghurt as compared to control sample. Gurkan et al. [32], who studied that some variations detected in pH values of the samples throughout 21 days. Apple polyphenols possessed several numbers of carboxyl and hydroxyl groups, water solubility and polarity [33]. The values of pH and ionic strength as well as the number of soluble solids of yoghurt, which therefore influenced the intermolecular and inter-particle bonds, precipitation of proteins, and product texture and viscosity during yoghurt production is due to the fortification of polyphenols [17,34]. The results showed that CTL had the low acidity 0.82, followed by AE1 0.79 while, AE5 had the high acidity 1.01, at day 1 then significantly (*p* < 0.05) increased to 1.09, 1.11 and 1.25 respectively for 21 days (Table 2). The results showed that the fortification of APPE slightly increased the acidity percentage of yoghurt as compared to control sample. Gurkan et al. [32] studied that few significant variations were observed in the acidity during 21 days of storage. A similar finding was observed by Ramchandran and Shah [35] in probiotic yoghurts.

### 3.4. Physical Characteristics of Probiotic Yoghurt

**Figure 2:**
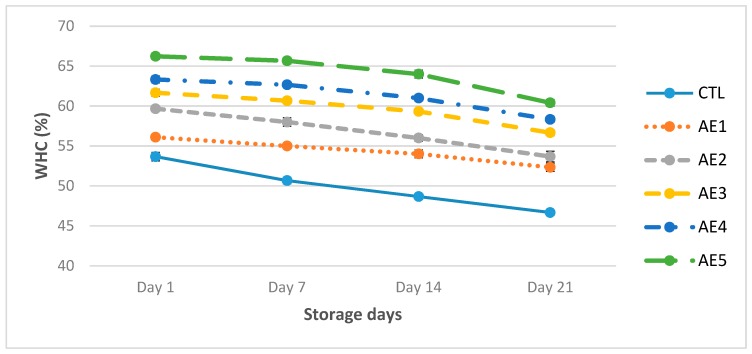
Water holding capacity of probiotic yoghurt with addition of APPE. CTL: Without APPE; AE_1_: 1% APPE; AE_2_: 2% APPE; AE_3_: 3% APPE; AE_4_: 4% APPE; AE_5_: 5% APPE.

**Figure 3:**
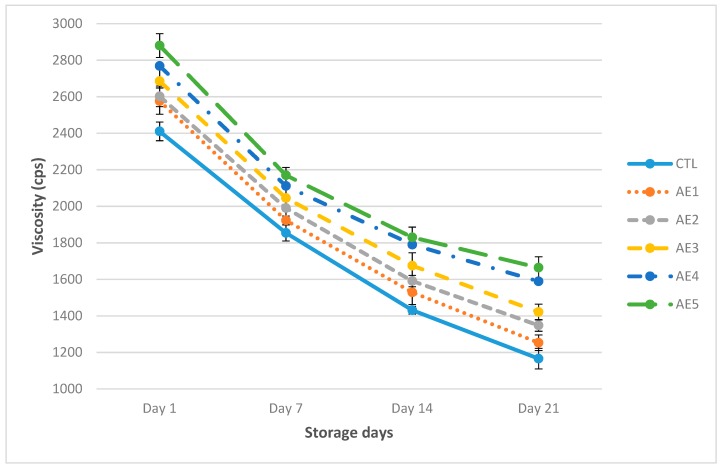
Viscosity of probiotic yoghurt with addition of APPE. CTL: Without APPE; AE_1_: 1% APPE; AE_2_: 2% APPE; AE_3_: 3% APPE; AE_4_: 4% APPE; AE_5_: 5% APPE.

The outcomes revealed that WHC of CTL was 53.67%, followed by AE1 56.10% while, the high WHC of AE5 was 66.23%, then significantly (*p* < 0.05) decreased to 46.67%, 52.33% and 60.41%, respectively, after 3 weeks (Figure 2). It was concluded that milk protein denaturation in yoghurt will enhance the WHC [36]. It was reported that denaturation of whey proteins enhanced the gelling characteristics with suitable heating and improved the surface area which allowed high water holding capacity of yoghurt [37]. The results revealed the viscosity of CTL was 2410 cp, followed by AE1 2576 cp, while AE5 had the high viscosity 2880 cp at 1st day which significantly (*p* < 0.05) decreased to 1166 cp, 1253 cp and 1664 cp, respectively, after 3 weeks (Figure 3). It was perceived that AE4 and AE5 revealed the high viscosity and WHC, whereas CTL and AE1 expressed low viscosity and WHC as matched to other yoghurt samples (Figure 2 and Figure 3). Results variation in WHC and viscosity of yoghurt might be due to total solids in yoghurt fortified with APPE. It was perceived that syneresis could be controlled by increasing total solid contents (14.44% to 14.96%) in probiotic yoghurt. It was perceived that the viscosity of probiotic yoghurt was increased by high total solid contents. Lucey and Singh [38] concluded that probiotic yoghurt with added APPE seemed to pour more slowly and more viscous than control yoghurt. Viscosity was influenced by the power and number of bonds between casein micelles of yoghurt, as well as their texture and spatial sharing. It was studied that the viscosity of yoghurt decreased during storage [39,40]. The viscosity of yoghurt affected by preparation structure together with the type of starter culture, type of stabilizer, processing methods and heat treatment [41]. It was studied that viscosity of yoghurt from buffalo milk was significantly (*p* < 0.05) reduced throughout storage [42].

### 3.5. Total Phenolic Contents (TPC) and Total Favonoid Content (TFC)

Free radicals and reactive oxygen are critical in various illnesses such as, arthritis, atherosclerosis and cancer [43]. The results presented that yoghurt fortified with APPE with concentration of 1%, 2%, 3% 4% and 5% significantly (*p* < 0.05) increased TPC (3.54, 4.76, 6.11, 7.45 and 8.94 g GAE/100 g of DW) respectively as compared to control yoghurt (1.48 g GAE/100g of DW) on 1 day. The results showed that TPC values were increased from day 1 to 2 weeks but decreased from two weeks to three weeks. The results showed that the total phenolic contents were statistically correlated to the total antioxidant activity of apple peels. Chinnici et al. [44] also compared the phenolic composition of apple peels to apple pulp, the total antioxidant capacity of apple peels was approximately 2.5 times more than that of apple pulp. Flavonoids are abundantly present in foods of plant origin, chalcones, flavones, isoflavonoids, flavanones, anthoxanthins and anthocyanins are the major classes of flavonoids. Flavonoids are very active scavengers of reactive oxygen species [45]. The TFC of probiotic yoghurt increased with different concentration 1%, 2%, 3%, 4% and 5% of APPE were as 0.59, 0.71, 0.97, 1.18, 1.35, respectively. Furthermore, the self-destruction of milk proteins resulted in the discharge of phenolic amino acids and non-phenolic compounds such as proteins and sugars which may inhibit throughout total phenolic evaluation [46].

### 3.6. Antioxidant Activity

Oxidative destruction can affect the flavor, bioactive compounds, destruction of nutrients, and generation of potentially toxic oxidation products [47]. APPE addition into fermented milk products led to the significant enhancement of antioxidant capacity while higher quantity of APPE corresponded to higher DPPH inhibition percentage (Table 3). The presence of APPE in probiotic yoghurt significantly increased (*p* < 0.05) the antioxidant activities in AE1, AE2, AE3, AE4 and AE5 compared to respective control yoghurt sample (27.13 ± 1.53%). Polyphenol compounds were antioxidants that had a positive effect on preventing oxidation [5].

### 3.7. Sensory Evaluation

**Figure 4:**
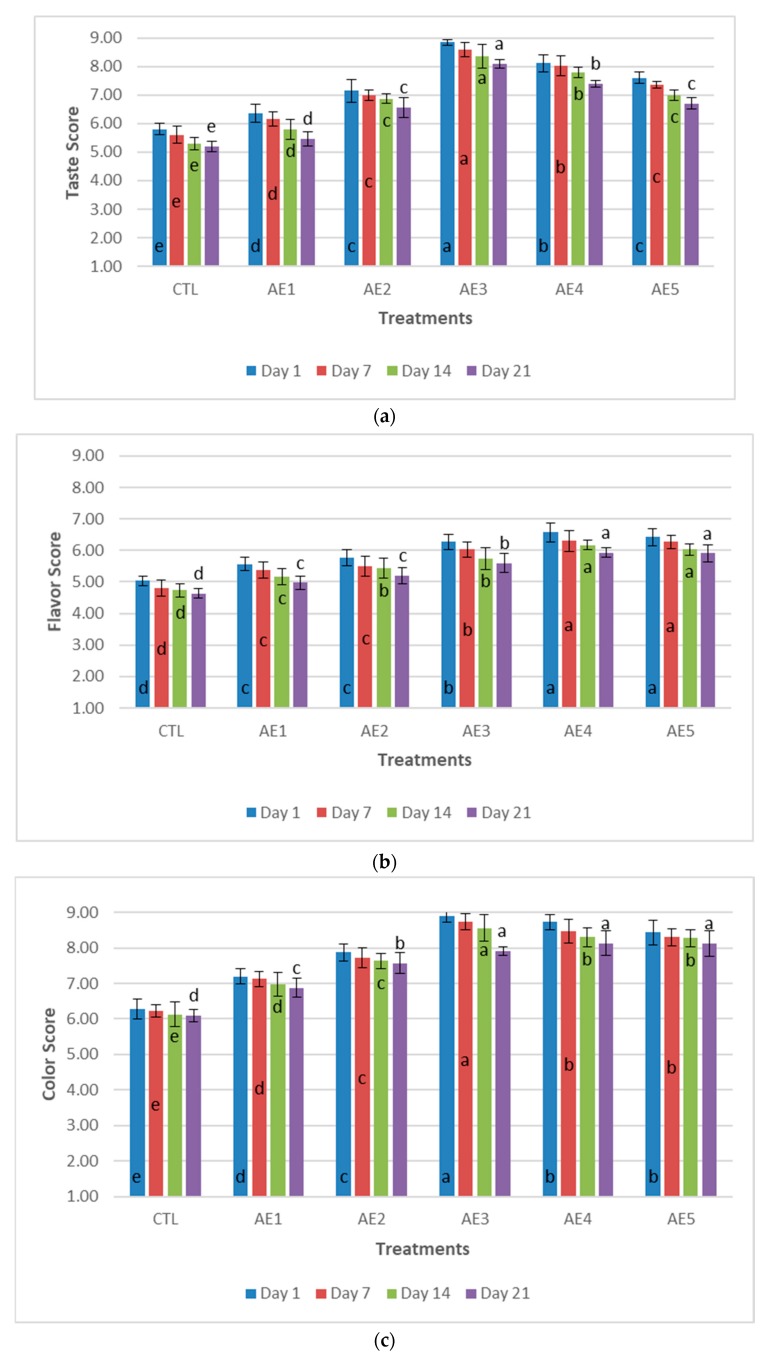

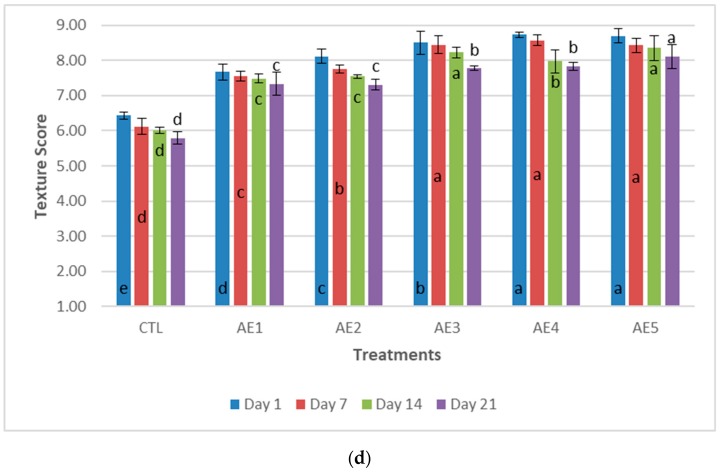
Sensory characteristics (**a**): taste attribute (**b**): flavor attribute (**c**): color attribute (**d**): texture attribute of probiotic yoghurt with addition of APPE. ^a,b,c,d,e^ Means in a column of same color with different superscripts were significantly different (*p* ≤ 0.05); CTL: Without APPE; AE_1_: 1% APPE; AE_2_: 2% APPE; AE_3_: 3% APPE; AE_4_: 4% APPE; AE_5_: 5% APPE.

Taste, flavor, color, and texture properties of probiotic yoghurt were evaluated as sensory attributes, shown in Figure 4. Sensory attributes ratings depended on the level of fortification. In the terms of taste and color, we found significant difference (*p* > 0.05) between the control and yoghurt enriched with APPE during storage. It was observed that AE3 sample got the best score of taste and color attributes as compared to others followed by the AE4, AE5, AE2 and AE1. The addition of APPE had a significant effect on the taste of probiotic yoghurt. The probiotic yoghurt samples were found to be sweeter and more aromatic by the panelists, due to the acidic and sweet taste of the apple peel extract. It was concluded that AE4 and AE5 samples had fruity flavor as compared to AE3, AE2 and AE1, while the lowest scores were obtained by the CTL significantly (Figure 4). The low score of all sensory aspects were found in the CTL sample. Texture was evaluated by both visual control and with a spoon or mouth feel. It was observed that AE5 sample had favorable texture as compare to other samples. The color attributes of yoghurt were influenced by the supplementation of herbs and fruits significantly [48]. A decrease in all sensory characteristics was detected during storage periods, probably due to proteolytic activity of bacteria and the production of acidity [49].

## 4. Conclusions

Yoghurt—fermented with *B. lactis* and *L. acidophilus* and fortified with APPE—proved to be an acceptable probiotic milk product. This APPE as prebiotic ingredient had viable counts greater than the minimum requirement to be considered probiotic. Notably, APPE had the highest viable counts throughout fermentation and 21 days of refrigerated storage. It was concluded that the survival of probiotics and antioxidant activity of yoghurt were enhanced with the addition of APPE. Probiotic yoghurt fortified with APPE can act as natural source of high-quality antioxidant and bioactive compounds. Nevertheless, addition of APPE did not lead to the substantial disruption of texture and flavor, showing an excellent sensory quality of probiotic yoghurt after 21 days of storage. 

Hence, it can be concluded that the addition of APPE in probiotic yoghurt gives a functional product with very good sensory and physical characteristics. Besides, this research has demonstrated that APPE could provide adequate protection of probiotic bacteria during production and storage of yoghurt. The present study provides grounds for further investigation on the possibility of using apple peels as a prebiotic for the survival of probiotic bacteria in food products, where it may be regarded as an alternative way of supplying probiotics to consumers.

## Figures and Tables

**Table 1:** Total viable count of bacteria in probiotic yoghurt with addition of APPE.

Viable Count	Treatment	Storage Period			
		Day 1	Day 7	Day 14	Day 21
*Streptococcus thermophilus* (log cfu/mL)	CTL	10.63 ± 0.49 ^Aa^	9.09 ± 0.02 ^Fb^	8.41 ± 0.06 ^Fc^	7.78 ± 0.05 ^Fd^
	AE_1_	10.50 ± 0.46 ^Aa^	9.45 ± 0.02 ^Eb^	9.02 ± 0.07 ^Ec^	8.83 ± 0.09 ^Ed^
	AE_2_	10.53 ± 0.50 ^Aa^	9.80 ± 0.05 ^Db^	9.42 ± 0.02 ^Dc^	9.05 ± 0.04 ^Dd^
	AE_3_	10.61 ± 0.34 ^Aa^	10.07 ± 0.03 ^Cb^	9.88 ± 0.09 ^Cc^	9.37 ± 0.05 ^Cd^
	AE_4_	10.65 ± 0.34 ^Aa^	10.35 ± 0.03 ^Bb^	10.08 ± 0.08 ^Bc^	9.86 ± 0.12 ^Bd^
	AE_5_	10.61 ± 0.15 ^Aa^	10.56 ± 0.07 ^Aa^	10.43 ± 0.02 ^Aa^	10.14 ± 0.11 ^Ab^
*Lactobacillus bulgaricus* (log cfu/mL)	CTL	10.24 ± 0.29 ^Aa^	9.10 ± 0.12 ^Fb^	8.67 ± 0.21 ^Fc^	7.60 ± 0.08 ^Fd^
	AE_1_	10.14 ± 0.45 ^Aa^	9.37 ± 0.04 ^Eb^	8.83 ± 0.20 ^Ec^	8.24 ± 0.02 ^Ed^
	AE_2_	10.22 ± 0.45 ^Aa^	9.55 ± 0.10 ^Db^	9.31 ± 0.01 ^Dc^	8.87 ± 0.09 ^Dd^
	AE_3_	10.35 ± 0.22 ^Aa^	9.89 ± 0.10 ^Cb^	9.69 ± 0.11 ^Cc^	9.30 ± 0.04 ^Cd^
	AE_4_	10.38 ± 0.20 ^Aa^	10.10 ± 0.06 ^Bb^	9.84 ± 0.17 ^Bc^	9.64 ± 0.10 ^Bd^
	AE_5_	10.37 ± 0.39 ^Aa^	10.34 ± 0.59 ^Aa^	10.29 ± 0.03 ^Aa^	10.01 ± 0.11 ^Ab^
*Lactobacillus acidophilus* (log cfu/mL)	CTL	11.14 ± 0.24 ^Aa^	9.85 ± 0.13 ^Fb^	8.67 ± 0.21 ^Fc^	8.07 ± 0.13 ^Fd^
	AE_1_	11.17 ± 0.15 ^Aa^	9.98 ± 0.10 ^Eb^	9.18 ± 0.10 ^Ec^	8.93 ± 0.13 ^Ed^
	AE_2_	11.22 ± 0.22 ^Aa^	10.09 ± 0.17 ^Db^	9.46 ± 0.02 ^Dc^	9.15 ± 0.05 ^Dd^
	AE_3_	11.24 ± 0.20 ^Aa^	10.37 ± 0.04 ^Cb^	10.03 ± 0.09 ^Cc^	9.64 ± 0.16 ^Cd^
	AE_4_	11.20 ± 0.30 ^Aa^	10.68 ± 0.18 ^Bb^	10.39 ± 0.05 ^Bc^	10.12 ± 0.07 ^Bd^
	AE_5_	11.16 ± 0.15 ^Aa^	11.08 ± 0.17 ^Aa^	10.77 ± 0.26 ^Ab^	10.41 ± 0.04 ^Ac^
*Bifidobacterium lactis* (log cfu/mL)	CTL	10.71 ± 0.17 ^Aa^	9.39 ± 0.03 ^Fb^	8.37 ± 0.10 ^Fc^	7.81 ± 0.15 ^Fd^
	AE_1_	10.68 ± 0.54 ^Aa^	9.79 ± 0.14 ^Eb^	9.42 ± 0.10 ^Ec^	9.19 ± 0.07 ^Ed^
	AE_2_	10.75 ± 0.10 ^Aa^	10.03 ± 0.08 ^Db^	9.80 ± 0.07 ^Dc^	9.60 ± 0.18 ^Dd^
	AE_3_	10.68 ± 0.59 ^Aa^	10.19 ± 0.11 ^Cb^	10.04 ± 0.05 ^Cc^	9.87 ± 0.12 ^Cd^
	AE_4_	10.76 ± 0.24 ^Aa^	10.40 ± 0.03 ^Bb^	10.18 ± 0.09 ^Bc^	10.05 ± 0.05 ^Bd^
	AE_5_	10.80 ± 0.41 ^Aa^	10.76 ± 0.13 ^Aa^	10.36 ± 0.23 ^Ab^	10.23 ± 0.05 ^Ac^

Values represent the mean ± standard deviation; n = 3. ^A,B,C,D,E,F^ Means in a column with different superscripts were significantly different (*p* ≤ 0.05). ^a,b,c,d^ Means in a row with different superscripts were significantly different (*p* ≤ 0.05). CTL: Without APPE; AE_1_: 1% APPE; AE_2_: 2% APPE; AE_3_: 3% APPE; AE_4_: 4% APPE; AE_5_: 5% APPE.

**Table 2:** Chemical characteristics of probiotic yoghurt with addition of APPE.

Composition	Treatment	Storage Period			
		Day 1	Day 7	Day 14	Day 21
pH	CTL	4.56 ± 0.01 ^Aa^	4.41 ± 0.03 ^Ab^	4.28 ± 0.03 ^Ac^	4.19 ± 0.02 ^Ad^
	AE_1_	4.51 ± 0.01 ^Aa^	4.40 ± 0.04 ^Ab^	4.24 ± 0.03 ^Ac^	4.13 ± 0.03 ^Ad^
	AE_2_	4.47 ± 0.03 ^Aa^	4.36 ± 0.04 ^Ab^	4.22 ± 0.01 ^Ac^	4.04 ± 0.02 ^Ad^
	AE_3_	4.46 ± 0.01 ^Aa^	4.33 ± 0.03 ^Ab^	4.14 ± 0.04 ^Ac^	4.03 ± 0.02 ^Ad^
	AE_4_	4.40 ± 0.02 ^Aa^	4.31 ± 0.06 ^Ab^	4.19 ± 0.03 ^Ac^	4.12 ± 0.05 ^Ad^
	AE_5_	4.36 ± 0.01 ^Aa^	4.33 ± 0.04 ^Aa^	4.14 ± 0.04 ^Ab^	4.02 ± 0.08 ^Ac^
Acidity (%)	CTL	0.82 ± 0.08 ^Aa^	0.92 ± 0.06 ^Aa^	1.06 ± 0.04 ^Ab^	1.09 ± 0.06 ^Ab^
	AE_1_	0.79 ± 0.06 ^Aa^	0.92 ± 0.07 ^Ab^	1.10 ± 0.02 ^Ac^	1.11 ± 0.04 ^Ac^
	AE_2_	0.90 ± 0.08 ^Aa^	1.00 ± 0.04 ^Aa^	1.15 ± 0.03 ^Ab^	1.18 ± 0.03 ^Ab^
	AE_3_	0.91 ± 0.06 ^Aa^	1.00 ± 0.06 ^Aa^	1.15 ± 0.04 ^Ab^	1.20 ± 0.03 ^Ab^
	AE_4_	0.95 ± 0.04 ^Aa^	1.05 ± 0.04 ^Aa^	1.18 ± 0.03 ^Ab^	1.22 ± 0.02 ^Ab^
	AE_5_	1.01 ± 0.03 ^Aa^	1.10 ± 0.02 ^Aa^	1.24 ± 0.02 ^Ab^	1.25 ± 0.04 ^Ab^
Total Solids (%)	CTL	14.48 ± 0.02 ^Ba^	14.45 ± 0.07 ^Ba^	14.47 ± 0.03 ^Ba^	14.44 ± 0.03 ^Ba^
	AE_1_	14.56 ± 0.06 ^Ba^	14.60 ± 0.05 ^Ba^	14.63 ± 0.03 ^Ba^	14.61 ± 0.03 ^Ba^
	AE_2_	14.66 ± 0.06 ^Aa^	14.70 ± 0.03 ^Aa^	14.73 ± 0.05 ^Aa^	14.76 ± 0.05 ^Aa^
	AE_3_	14.78 ± 0.09 ^Aa^	14.79 ± 0.02 ^Aa^	14.84 ± 0.08 ^Aa^	14.86 ± 0.04 ^Aa^
	AE_4_	14.77 ± 0.22 ^Aa^	14.82 ± 0.07 ^Aa^	14.85 ± 0.01 ^Aa^	14.86 ± 0.03 ^Aa^
	AE_5_	14.79 ± 0.11 ^Aa^	14.82 ± 0.02 ^Aa^	14.96 ± 0.02 ^Aa^	14.93 ± 0.08 ^Aa^

Values represent the mean ± standard deviation; n = 3. ^a,b,c,d^ Means in a row with different superscripts were significantly different (*p* ≤ 0.05). ^A,B,C^ Means in a column with different superscripts were significantly different (*p* ≤ 0.05). CTL: Without APPE; AE_1_: 1% APPE; AE_2_: 2% APPE; AE_3_: 3% APPE; AE_4_: 4% APPE; AE_5_: 5% APPE.

**Table 3:** Total phenolic contents of probiotic yoghurt with addition of APPE.

Composition	Treatment	Storage Period			
		Day 1	Day 7	Day 14	Day 21
Total Phenolic Contents (g GAE/100g DW)	CTL	1.48 ± 0.91 ^Fc^	2.21 ± 0.17 ^Fb^	2.46 ± 0.06 ^Fa^	2.10 ± 0.16 ^Eb^
	AE_1_	3.54 ± 0.08 ^Ec^	3.97 ± 0.08 ^Eb^	4.34 ± 0.05 ^Ea^	4.01 ± 0.04 ^Db^
	AE_2_	4.76 ± 0.45 ^Dc^	5.23 ± 0.05 ^Db^	5.86 ± 0.15 ^Da^	5.54 ± 0.21 ^Ca^
	AE_3_	6.11 ± 0.20 ^Cc^	6.73 ± 0.17 ^Cb^	7.34 ± 0.17 ^Ca^	7.06 ± 0.32 ^Ba^
	AE_4_	7.45 ± 0.12 ^Bc^	8.04 ± 0.11 ^Bb^	8.85 ± 0.23 ^Ba^	8.63 ± 0.28 ^Ba^
	AE_5_	8.94 ± 0.16 ^Ab^	9.35 ± 0.16 ^Aa^	9.71 ± 0.21 ^Aa^	9.52 ± 0.17 ^Aa^
Total Flavonoid Contents (g QE/100g DW)	CTL	0.07 ± 0.09 ^Ea^	0.07 ± 0.31 ^Da^	0.06 ± 0.34 ^Da^	0.04 ± 0.13 ^Da^
	AE_1_	0.59 ± 0.06 ^Da^	0.55 ± 0.20 ^Ca^	0.52 ± 0.04 ^Ca^	0.48 ± 0.15 ^Ca^
	AE_2_	0.71 ± 0.16 ^Ca^	0.66 ± 0.18 ^Ca^	0.61 ± 0.07 ^Ca^	0.59 ± 0.21 ^Ca^
	AE_3_	0.97 ± 0.30 ^Ca^	0.93 ± 0.15 ^Ba^	0.89 ± 0.16 ^Ba^	0.84 ± 0.14 ^Ba^
	AE_4_	1.18 ± 0.24 ^Ba^	1.14 ± 0.12 ^Aa^	1.09 ± 0.13 ^Ba^	1.04 ± 0.13 ^Aa^
	AE_5_	1.35 ± 0.15 ^Aa^	1.29 ± 0.07 ^Aa^	1.23 ± 0.31 ^Aa^	1.18 ± 0.11 ^Aa^
DPPH Inhibition (%)	CTL	27.13 ± 1.53 ^Ca^	24.67 ± 2.08 ^Db^	29.21 ± 0.33 ^Da^	27.12 ± 0.58 ^Ca^
	AE_1_	39.21 ± 3.30 ^Ba^	34.70 ± 1.00 ^Ca^	28.21 ± 1.00 ^Db^	26.83 ± 1.53 ^Cb^
	AE_2_	46.67 ± 1.53 ^Ba^	41.30 ± 2.65 ^Ba^	36.70 ± 4.00 ^Cb^	31.28 ± 3.21 ^Bb^
	AE_3_	51.27 ± 1.15 ^Ba^	47.97 ± 1.15 ^Ba^	42.53 ± 1.53 ^Bb^	37.02 ± 2.08 ^Bb^
	AE_4_	59.96 ± 2.52 ^Aa^	55.67 ± 1.53 ^Aa^	49.65 ± 1.00 ^Bb^	42.03 ± 4.04 ^Ab^
	AE_5_	67.10 ± 2.00 ^Aa^	61.77 ± 0.58 ^Ab^	58.31 ± 0.58 ^Ab^	47.02 ± 2.31 ^Ac^

Values represent the mean ± standard deviation; n = 3. ^a,b,c^ Means in a row with different superscripts were significantly different (*p* ≤ 0.05). ^A,B,C,D,E,F^ Means in a column with different superscripts were significantly different (*p* ≤ 0.05). CTL: Without APPE; AE_1_: 1% APPE; AE_2_: 2% APPE; AE_3_: 3% APPE; AE_4_: 4% APPE; AE_5_: 5% APPE.
